# Molecular basis for the substrate specificity and catalytic mechanism of thymine-7-hydroxylase in fungi

**DOI:** 10.1093/nar/gkv979

**Published:** 2015-10-01

**Authors:** Wenjing Li, Tianlong Zhang, Jianping Ding

**Affiliations:** 1 National Center for Protein Science Shanghai, State Key Laboratory of Molecular Biology, Institute of Biochemistry and Cell Biology, Shanghai Institutes for Biological Sciences, Chinese Academy of Sciences, 320 Yue-Yang Road, Shanghai 200031, China; 2 Collaborative Innovation Center for Genetics and Development, Fudan University, 2005 Song-Hu Road, Shanghai 200438, China

## Abstract

TET proteins play a vital role in active DNA demethylation in mammals and thus have important functions in many essential cellular processes. The chemistry for the conversion of 5mC to 5hmC, 5fC and 5caC catalysed by TET proteins is similar to that of T to 5hmU, 5fU and 5caU catalysed by thymine-7-hydroxylase (T7H) in the nucleotide anabolism in fungi. Here, we report the crystal structures and biochemical properties of *Neurospora crassa* T7H. T7H can bind the substrates only in the presence of cosubstrate, and binding of different substrates does not induce notable conformational changes. T7H exhibits comparable binding affinity for T and 5hmU, but 3-fold lower affinity for 5fU. Residues Phe292, Tyr217 and Arg190 play critical roles in substrate binding and catalysis, and the interactions of the C5 modification group of substrates with the cosubstrate and enzyme contribute to the slightly varied binding affinity and activity towards different substrates. After the catalysis, the products are released and new cosubstrate and substrate are reloaded to conduct the next oxidation reaction. Our data reveal the molecular basis for substrate specificity and catalytic mechanism of T7H and provide new insights into the molecular mechanism of substrate recognition and catalysis of TET proteins.

## INTRODUCTION

Methylation at the C5 position of cytosine in DNA, which mainly exists in the CpG dinucleotide context in mammals, is an important epigenetic modification. Proper establishment and maintenance of the DNA methylation patterns are crucial for many important cellular processes, including embryonic development, genomic imprinting, X-chromosome inactivation and chromosome stability (1–4). The ten-eleven translocation (TET) proteins are a group of Fe^2+^ and α-ketoglutarate (α-KG) dependent dioxygenases which can catalyse the conversion of 5-methylcytosine (5mC) to 5-hydroxymethylcytosine (5hmC), 5-formylcytosine (5fC) and 5-carboxylcytosine (5caC) in DNA (5–9). The final product 5caC can be recognized and excised by thymine-DNA glycosylase (TDG) and subsequently substituted by unmodified cytosine via base excision repair (BER) pathway (9,10). This process is currently believed to be an active DNA demethylation pathway. Thus, the TET proteins are proposed to play critical roles in epigenetic regulation (11–14), and aberrant expression, abnormal regulation and mutations of TET proteins are associated with many serious human diseases including cancers (13,15).

The crystal structures of the catalytic domain of *Homo sapiens* TET2 (HsTET2) and the full-length *Naegleria gruberi* TET-like dioxygenase 1 (NgTET1) in complexes with 5mC-containing DNA have been reported recently (16,17). In these structures, the base to be oxidized is flipped out of the double-stranded DNA (dsDNA) and inserted into the active site of HsTET2 or NgTET1. In the HsTET2-DNA and NgTET1-DNA complexes, the C5-methyl group of 5mC is not specifically recognized by any residues but has van der Waals contacts with the C1-carboxylate of NOG and the side chains of two surrounding residues (16,17). Nevertheless, how the TET proteins recognize or distinguish different modification groups on the C5 position of cytosine and catalyse the consecutive oxidations of 5mC to 5hmC, 5fC and 5caC are not well understood.

Intriguingly, the chemical characteristics of the successive oxidations of 5mC to 5hmC, 5fC and 5caC in mammals share great similarities with that of the successive oxidations of thymine (T or 5-methyluracil) to 5-hydroxymethyluracil (5hmU), 5-formyluracil (5fU) and 5-carboxyluracil (5caU), an essential process in the thymidine salvage pathway of nucleotide anabolism in some fungi. The thymidine salvage pathway, which was first discovered in *Neurospora crassa*, consists of a series of catalytic reactions (18). After the hydrolysis of thymine ribonucleoside to ribose and thymine (19), the consecutive oxidations of thymine to 5hmU, 5fU and 5caU are catalysed by thymine-7-hydroxylase (T7H) (20–23), and the subsequent decarboxylation of 5caU to U is catalysed by isoorotate decarboxylase (IDCase) (24–26). Like the TET proteins, T7H is also a Fe^2+^ and α-KG dependent dioxygenase (23). Previous biochemical studies have characterized the enzymatic properties and revealed some insights into the catalytic mechanism of T7H (27–29). However, the structural and molecular basis for the substrate recognition and binding and the catalytic mechanism of T7H remains elusive.

Here we report the crystal structures of *N. crassa* T7H (NcT7H) in apo form, in α-KG-bound form and in complexes with α-KG and T, 5hmU or 5fU. Our structural and biochemical data together reveal the molecular basis for how T7H recognizes different substrates and catalyses the consecutive oxidations and provide new insights into the molecular mechanism of the substrate recognition and catalysis of the TET proteins.

## MATERIALS AND METHODS

### Cloning, expression and purification

The genes encoding the full-length NcT7H (residues 1–333) and the C-terminal truncated NcT7H (residues 1–299, NcT7HΔC) were amplified by polymerase chain reaction from the genomic DNA of *N. crassa*. Each gene was cloned into the pET-28a vector (Novagen) with a His_6_-tag at the C-terminus. The constructed plasmids were transformed into *Escherichia coli* BL21(DE3) Condon Plus strain (Novagen). The bacterial cells were grown in LB medium supplemented with 0.05 mg/ml kanamycin at 37°C to OD_600_ of 0.6, and then induced with 0.2 mM IPTG at 16°C overnight. The cells were collected, resuspended and lysed on ice by sonication in the lysis buffer (20 mM Tris-HCl, pH 8.0, 150 mM NaCl, 5% glycerol, 2 mM β-mercaptoethanol, and 1 mM phenylmethylsulfonyl fluoride). The cell debris was precipitated by centrifugation at 18 000 g, and the supernatant was collected for protein purification.

The proteins were purified by affinity chromatography using a Ni-NTA column (Qiagen) with the storage buffer (20 mM Tris-HCl, pH 8.0, and 150 mM NaCl) supplemented with 20 mM imidazole and 250 mM imidazole serving as the washing buffer and the elution buffer, respectively. The proteins were further purified with gel filtration using a Superdex G75 16/60 column (GE Healthcare). Expression and purification of the Se-Met substituted NcT7HΔC followed the same procedures as the native protein except that the bacterial cells were grown in M9 medium containing amino acids Lys, Thr, Phe, Leu, Ile, Val, Se-Met, and 1% lactose. Constructs of the NcT7H mutants containing point mutations were generated using the QuikChange Site-Directed Mutagenesis kit (Stratagene) and verified by sequencing. Expression and purification of the mutants were the same as the wild-type protein. The purified proteins were of high purity (above 95%) as analysed by SDS-PAGE.

### Crystallization, data collection and structure determination

Crystallization was performed using the sitting drop vapour diffusion method at 16°C. Crystals of Se-Met NcT7HΔC in apo form were grown in drops consisting of an equal volume of the protein solution (30 mg/ml) and the reservoir solution [0.2 M (NH_4_)_2_SO_4_, 0.1 M MES, pH 6.5, and 30% (w/v) PEGMME 5000]. In order to obtain catalytically inactive structures of NcT7H in complexes with different substrates, the protein solution was supplemented with 2 mM NiCl_2_ to mimic Fe^2+^. Crystals of NcT7H in complex with α-KG were grown in drops consisting of the protein solution supplemented with α-KG (1:4 molar ratio) and the reservoir solution (0.1 M BIS-TRIS, pH 5.5, and 25% (w/v) PEG 3,350). Crystals of NcT7H in complexes with α-KG and T, 5hmU or 5fU (Sigma) were grown in drops containing the protein solution supplemented with α-KG and the substrate (1:4:8 molar ratio) and the same reservoir solution. The crystals were cryoprotected using the reservoir solution supplemented with 25% ethylene glycol and then flash-cooled into liquid N_2_. Diffraction data were collected at 100 K at BL17U of Shanghai Synchrotron Radiation Facility and BL19U1 of National Facility for Protein Science Shanghai, and processed with HKL2000 (30). The statistics of the diffraction data are summarized in Table 1.

**Table 1.** Summary of diffraction data and refinement statistics

	NcT7HΔC	NcT7H-AKG	NcT7H-T	NcT7H-5hmU	NcT7H-5fU
**Data collection**					
Wavelength (Å)	0.9792	0.9792	0.9792	0.9792	0.9199
Space group	*P*6_3_	*P2_1_*	*P2_1_*	*P2_1_*	*P2_1_*
Cell dimensions					
*a* (Å)	119.1	57.2	56.9	56.8	56.8
*b* (Å)	119.1	154.7	156.3	155.8	155.6
*c* (Å)	56.6	75.4	76.0	76.0	76.0
α (°)	90	90	90	90	90
β (°)	90	90.6	91.8	91.7	91.7
γ (°)	120	90	90	90	90
Resolution (Å)	50.0–2.30 (2.38–2.30)^a^	50.0–2.10 (2.18–2.10)	50.0–2.05 (2.12–2.05)	50.0–2.35 (2.43–2.35)	50.0–2.15 (2.23–2.15)
Observed reflections	217 111	249 555	314 882	166 072	290 491
Unique reflections (*I*/σ(*I*) > 0)	20 591 (2062)	75 367 (7543)	82 717 (8246)	53 781 (5427)	69 438 (6848)
*I*/σ(*I*)	29.2 (5.0)	17.4 (3.2)	16.6 (3.0)	19.3 (10.2)	16.1 (3.7)
Completeness (%)	100.0 (100.0)	99.3 (99.9)	99.9 (100.0)	98.3 (99.4)	98.5 (97.1)
Redundancy	10.5 (10.6)	3.3 (3.3)	3.8 (3.8)	3.1 (3.1)	4.2 (4.1)
*R*_merge_ (%) ^b^	10.2 (54.0)	7.9 (40.6)	8.5 (46.8)	6.9 (12.6)	12.3 (59.4)

**Refinement**					
Resolution (Å)	50.0–2.30	50.0–2.10	50.0–2.05	50.0–2.35	50.0–2.15
No. reflections					
Working set	19 527	71 601	78 516	51 062	66 062
Test set	1053	3731	4130	2689	3343
*R*_work_/*R*_free_ (%) ^c^	19.6 / 22.4	18.5 / 22.2	18.4 / 21.5	18.1 / 22.5	18.4 / 22.8
No. atoms					
Protein	2049	10 138	10 183	10 140	10 145
Cosubstrate	-	40	40	40	40
Substrate	-	-	36	40	40
Water	109	495	593	557	569
B-factors					
Protein	50.4	46.5	35.8	30.7	28.0
Cosubstrate	-	49.2	30.0	26.2	25.0
Substrate	-	-	37.1	39.7	34.9
Ion	32.7	43.4	27.7	22.3	22.1
Water	54.0	54.6	40.1	32.7	32.0
R.m.s. deviations					
Bond lengths (Å)	0.005	0.006	0.005	0.005	0.006
Bond angles (°)	1.1	1.1	1.0	1.0	1.1
Ramachandran plot (%)					
Most favoured	92.3	92.0	91.8	91.4	91.4
Allowed	7.2	7.7	7.9	8.2	8.3
Generously allowed	0.5	0.3	0.4	0.4	0.4

^a^Numbers in parentheses represent the highest resolution shell.

^b^*R*_merge_ = ∑_hkl_∑_i_|I_i_(hkl)−<I(hkl)>|/∑_hkl_∑_i_I_i_(hkl).

^c^*R* = ∑_hkl_∥F_o_|−|F_c_∥/∑_hkl_|F_o_|.

The apo NcT7HΔC structure was solved at 2.3 Å resolution using the single-wavelength anomalous dispersion (SAD) method as implemented in Phenix (31), which identified 4 Se atoms with a figure-of-merit of 0.40 and produced an interpretable electron density map. All of the other NcT7H structures were solved by the molecular replacement (MR) method using the apo NcT7HΔC structure as the search model. Model building was performed with Coot (32) and structure refinement was carried out using Phenix (31) and Refmac5 (33). Structural analysis was carried out using programs in CCP4 (34). The stereochemical geometry and quality of the structure models were analysed using Procheck (35). All graphics were generated using Pymol (www.pymol.org). The statistics of the refinement and the final structure models are summarized in Table 1.

### Enzymatic activity assay

The apparent enzymatic activities of the wild-type and mutant NcT7H and the wild-type NcT7HΔC to catalyse the consecutive oxidations of T to 5hmU, 5fU and 5caU were analysed using the HPLC method, which was modified based on the method used in the enzymatic activity assay of IDCase (26). The reaction solution (100 μl) consisted of 5 μM (for T and 5hmU) or 2 μM (for 5fU) enzyme, 2 mM α-KG, and 2 mM substrate in the reaction buffer [20 mM K_3_PO_4_, 1 mM (NH_4_)_2_Fe(SO_4_)_2_, and 2 mM ascorbic acid]. The reaction was carried out at 37ºC for 1 h and then stopped by heating the solution to 100ºC for 3 min. The reaction mixture was analysed using an Agilent 1200 HPLC instrument (Agilent Technologies) with an AQ-C18 column (5 μm particle size, 25 cm × 4.6 mm). The mobile phase was 20 mM NH_4_OAc (pH 3.5) running at the rate of 0.5 ml/min, and the detector was set at 260 nm. Standard T, 5hmU, 5fU, and 5caU were used as references. T, 5hmU, 5fU, and 5caU have distinct retention times and their contents in the reaction mixture were semi-quantified based on integrations of the respective retention peaks. The apparent enzymatic activity was represented by the fraction of the substrate oxidized.

To determine the kinetic parameters of T, 5hmU or 5fU oxidation by NcT7H, we used the SCS/PK/LDH-coupled system to monitor the production of succinate (36). Reaction was carried out at 25ºC in a buffer containing 100 mM Hepes (pH 7.5), 1 mM α-KG, 0.2 mM ascorbic acid, 0.2 mM (NH_4_)_2_Fe(SO_4_)_2_, 0.1 mM MgCl_2_, 0.4 mM ATP, 0.4 mM coenzyme A (CoA), 1 mM phosphoenolpyruvate, 0.4 mM NADH and varied concentrations of substrate (T, 5hmU or 5fU). The coupling enzymes succinyl-CoA synthetase (SCS, 5 μM, expressed and purified as His_6_-tag fused protein) and pyruvate kinase (PK)/lactate dehydrogenase (LDH) (Sigma, 6–10 units of PK and 9–14 units of LDH), along with the wild-type or mutant NcT7H (1–20 μM), were added to the reaction mixture to a final volume of 500 μl. The reaction was initiated by addition of the wild-type or mutant NcT7H, and the enzymatic activity was measured by monitoring the rate of NADH oxidation, which has an extinction coefficient of 6220 M^−1^cm^−1^ at 340 nm, using a Beckman DU800 spectrophotometer (Beckman Coulter). All kinetic data were fitted to the Michaelis–Menten equation using Prism 5.0 (Graphpad Software). All experiments were performed in triplicates.

### Isothermal titration calorimetry analysis

The thermodynamic parameters of the wild-type and mutant NcT7H with different ligands were measured using the isothermal titration calorimetry (ITC) method with an ITC200 Micro-calorimeter (MicroCal) at 25ºC. Prior to titrations with the substrates, NcT7H was incubated with NOG or succinate at 1:2 molar ratio. In all experiments, the initial injection of 0.4 μl of the ligand solution was discarded to eliminate the effect of titrant diffusion across the syringe tip during the equilibration process, and each dataset consisted of 20 injections of 2 μl each of 2 mM α-KG, NOG, succinate, U, T, 5hmU, 5fU or 5caU into the sample cell containing 250 μl of 0.2 mM NcT7H. The heats of dilution were negligible in all cases. Binding constants and other thermodynamic parameters were determined by fitting the integrated titration data using a single binding site model by a nonlinear least-squares method implemented in MicroCal Origin software version 7.0.

## RESULTS

### Overall structure of NcT7H

Crystallization of the full-length NcT7H (residues 1–333) alone yielded no crystal. Crystallization of a C-terminal truncated NcT7H (residues 1–299, NcT7HΔC) alone yielded the apo-form crystals. Crystallization of the full-length NcT7H in the presence of α-KG or α-KG and the substrate (T, 5hmU or 5fU) yielded crystals of the α-KG-bound or the substrate-bound complexes, respectively. The Crystal structure of the apo NcT7HΔC was solved by the SAD method at 2.3 Å resolution (Table 1). The asymmetric unit contains one NcT7HΔC molecule, which is well defined except four surface exposed regions (residues 95–116, residues 198–207, residues 278–282, and residues 296–299). There is a metal ion bound at the active site, which is provisionally interpreted as calcium as the ICP-AES (inductively coupled plasma atomic emission spectroscopy) analysis revealed that calcium is the most abundant metal in the protein solution (Supplementary Table S1) and the structure refinement yielded a reasonable B-factor (Table 1). Since no divalent metal ion was added in the purification and the crystallization solution, the bound Ca^2+^ is presumably co-purified with the enzyme.

The crystal structures of the full-length NcT7H in complex with α-KG (NcT7H-AKG), and in complexes with α-KG and T (NcT7H-T), with α-KG and 5hmU (NcT7H-5hmU), and with α-KG and 5fU (NcT7H-5fU) were solved by the MR method at 2.1 Å, 2.05 Å, 2.35 Å, and 2.15 Å resolution, respectively (Table 1). In all these structures, the asymmetric unit contains four NcT7H molecules, of which two molecules consist of the whole polypeptide chain and the other two have a disordered region (approximately residues 94–114) due to differed crystal packing environments. Comparison of the four NcT7H molecules shows no significant conformational differences (RMSD of <0.6 Å for all Cα atoms). At the active site, α-KG and the substrate are well defined with evident electron density (Supplementary Figure S1). In addition, similar to the apo NcT7HΔC structure, a metal ion is bound at the active site, which is assigned as Ni^2+^ owing to the presence of NiCl_2_ in the crystallization solution and the verification of the ICP-AES analysis (Supplementary Table S1).

**Figure 1.**
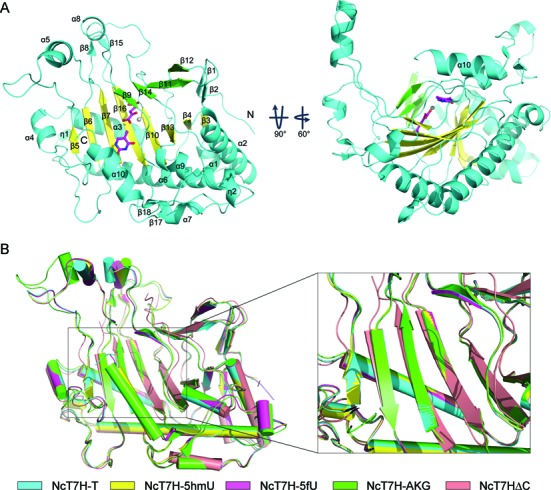
Overall structure of NcT7H. (**A**) Overall structure of NcT7H in complex with α-KG and T. The minor and major β-sheets that constitute the DSBH fold are coloured in green and yellow, respectively. Other structure elements flanking the DSBH core are shown in cyan. The location of the active site is indicated with a metal ion shown as a grey sphere, the cosubstrate α-KG and the substrate T shown as ball-and-stick models and coloured in magenta. The secondary structure elements are labeled. (**B**) Comparison of the overall structures of NcT7HΔC (salmon), NcT7H-AKG (green), NcT7H-T (cyan), NcT7H-5hmU (yellow) and NcT7H-5fU (magenta).

Similar to other α-KG dependent dioxygenases (37), NcT7H adopts a variation of double-stranded β-helix (DSBH) fold as the core (Figure 1A and Supplementary Figure S2). The major β-sheet of the DSBH fold comprises of eight β-strands (β3–7, β10, β13, and β16), and the minor β-sheet comprises of four β-strands (β9, β11–12, and β14). In addition, two short β-strands (β1 and β2) are located at one end (called the back end) of the DSBH core, and eight α-helices wrap around the DSBH core. These structure elements together form the compact globular catalytic domain. Besides, there are two large insertions (one between β5 and β6 and the other between β7 and β9) which form several extra structure elements flanking the catalytic domain (Figure 1A and Supplementary Figure S2).

### Structure of the active site

The active site of NcT7H is located at the front end (opposite to the back end) of the DSBH core and partially covered by the α10 helix at the C-terminus (Figure 1A). It is composed of residues mainly from strands β6, β7, β9, β10, β14, and β16. Structural comparison shows that the α-KG binding does not cause significant conformational changes in the overall structure (RMSD of <1.0 Å for all Cα atoms) but induces small conformational changes at the active site (Figure 1B and Supplementary Figure S3A). Compared to the apo NcT7HΔC structure, in the α-KG-bound NcT7H structure, the residues composing the active site and the associated β-strands move slightly towards α-KG, and the active site assumes a relatively more compact conformation. In addition, the binding of the substrate also does not cause obvious conformational changes at the active site among different substrate-bound NcT7H structures as well as compared to the α-KG-bound NcT7H structure (Supplementary Figure S3B–F). However, the lack of major conformational changes at the active site of NcT7H upon α-KG and substrate binding could be due to the crystalline state, which might not exactly reflect the solution situation of the enzyme. For example, the NMR and crystallographic studies of the dioxygenase AlkB have shown that the active site of AlkB is conformationally flexible in solution and the cosubstrate and substrate binding may induce substantial conformational changes at the active site (38,39).

**Figure 2.**
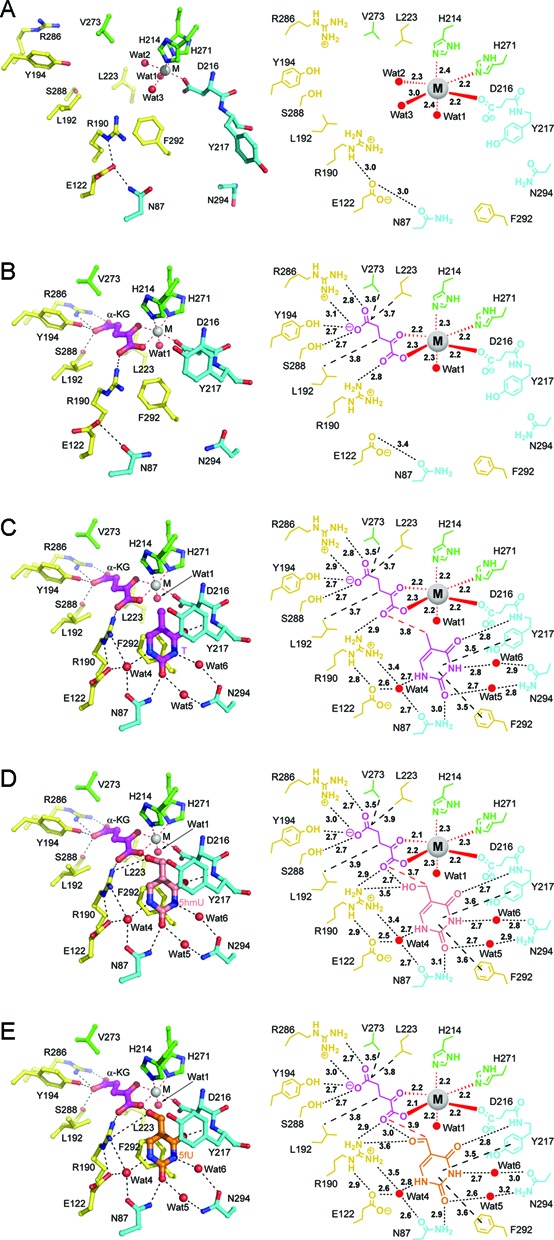
Structure (left panel) and schematic diagram (right panel) of the active site showing the interactions of the metal ion, α-KG and the substrate with the surrounding residues. (**A**) The apo NcT7HΔC. (**B**) The NcT7H-AKG complex. (**C**) The NcT7H-T complex. (**D**) The NcT7H-5hmU complex. (**E**) The NcT7H-5fU complex. The substrate, α-KG and the residues are shown with ball-and-stick models, the metal ion with a grey sphere, and the water molecules with red spheres. The colour coding for the residues is the same as in Figure 1A. The substrates T, 5hmU and 5fU are coloured in magenta, salmon and orange, respectively. The coordination bonds of the metal ion are shown with red dotted lines, the hydrogen bonds with black dotted lines, and the key hydrophobic interactions with black dashed lines. The bond lengths (Å) are indicated.

The active site of NcT7H is consisted of the binding sites for the metal ion, the cosubstrate and the substrate. In the apo NcT7HΔC structure, the Ca^2+^ is coordinated by three strictly conserved residues His214, Asp216 and His271, which constitute the characteristic HXD/E…H motif of α-KG dependent dioxygenases (37,40), and three water molecules in an octahedral geometry (Figure 2A and Supplementary Figure S4). In the ligand-bound NcT7H structures, the Ni^2+^ retains the coordinations with the three residues and one water molecule (Wat1), but the other two water molecules are replaced by the C1-carboxylate and C2-oxo groups of α-KG (Figure 2B–E). The binding and coordination geometry of the Ca^2+^ and Ni^2+^ may resemble these of the Fe^2+^ to some extent (41).

In the ligand-bound NcT7H structures, α-KG binds to a positively charged pocket with the C5-carboxylate embedded in the pocket and the C1-carboxylate oriented towards the substrate (Figure 2B–E and Supplementary Figure S5). The C5-carboxylate is stabilized by four hydrogen-bonding interactions with the side chains of Tyr194, Arg286 and Ser288. The C1-carboxylate forms a hydrogen bond with the side chain of Arg190. In addition, several hydrophobic residues (including Leu192, Leu223 and Val273) are also involved in interactions with α-KG. Sequence alignment shows that all the residues involved in α-KG binding are strictly conserved in T7Hs from different species (Supplementary Figure S4). The interactions of α-KG with NcT7H are very similar to those observed in many other α-KG dependent dioxygenases (39,40,42,43).

In the substrate-bound NcT7H structures, T, 5hmU or 5fU binds to a pocket adjacent to the metal ion and α-KG, and has largely hydrophobic interactions and a few hydrophilic interactions with the surrounding residues (Figure 2C–E and Supplementary Figure S5B–D). Specifically, in the NcT7H-T complex, the uracil moiety of T makes π–π stacking interaction with the side chain of Phe292 and is covered on top in part by the side chain of Tyr217 via hydrophobic interactions (Figure 2C). In addition, the N1 atom of the uracil moiety makes indirect hydrogen bonds with the side chains of Asn87, Glu122 and Arg190 via a water molecule; the O2 group makes a direct hydrogen bond with the side chain of Asn87 and an indirect hydrogen bond with the side chain of Asn294 via a water molecule; the N3 atom makes an indirect hydrogen bond with the side chain of Asn294 via a water molecule; and the O4 group makes a direct hydrogen bond with the main-chain amine of Tyr217 (Figure 2C). Interestingly, the C5-methyl group (C51 atom) of T is not specifically recognized by any residues but has van der Waals contacts with the C1-carboxylate of α-KG and the side chains of Asp216 and His214 (about 3.6–3.9 Å) (Figure 2C), which is similar to 5mC in the HsTET2 and NgTET1 structures (16,17).

In the NcT7H-5hmU and NcT7H-5fU complexes, the uracil moiety of 5hmU or 5fU maintains almost identical interactions with the surrounding residues as that in the NcT7H-T complex (Figure 2C–E). The C51 atom of 5hmU and 5fU is located about 3.7 Å and 3.9 Å away from the C1-carboxylate of α-KG, respectively. In addition, the 5-hydroxymethyl group of 5hmU or the 5-formyl group of 5fU forms a hydrogen bond with the C1-carboxylate of α-KG and a hydrogen bond with the side chain of Arg190 (Figure 2D and E). In all the substrate-bound NcT7H structures, Arg190 is located at the entrance to the active site and its side chain makes hydrogen bonds with both α-KG and the substrate, suggesting that Arg190 might play a critical role in the recognition and binding of the cosubstrate and substrate and the catalytic reaction. Sequence alignment shows that all the residues involved in the substrate binding are strictly or highly conserved in T7Hs from different species (Supplementary Figure S4).

### Binding affinity of NcT7H for different substrates

**Figure 3.**
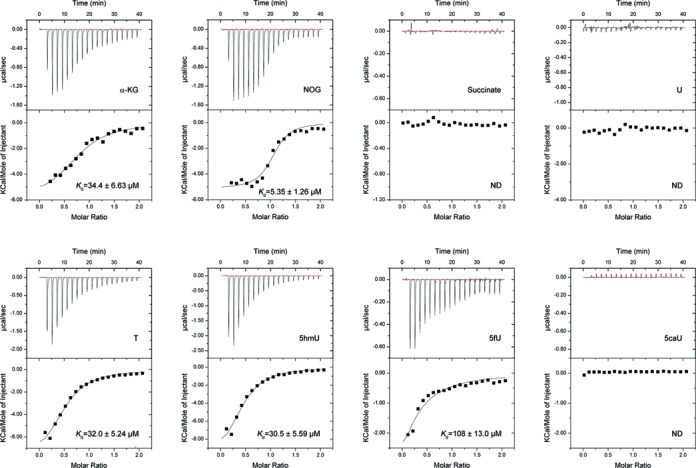
ITC analyses of the binding affinities of NcT7H towards different ligands. The binding affinities of NcT7H for α-KG, NOG and succinate and the binding affinities of NcT7H for U, T, 5hmU, 5fU and 5caU in the presence of NOG were analysed. The dissociation constant (*K*_d_) is indicated. ND, not detected.

NcT7H can catalyse the consecutive oxidations of T to 5hmU, 5fU and 5caU, which differ only at the C5 position of the uracil moiety. To investigate whether NcT7H has varied binding affinities for different substrates, we performed ITC experiments to analyse the binding affinities of NcT7H for α-KG and the substrates. NcT7H has a moderate binding affinity for α-KG (*K*_d_ of 34.4 ± 6.63 μM) and a 6-fold higher affinity for the α-KG analogue NOG (*K*_d_ of 5.35 ± 1.26 μM) (Figure 3 and Table 2), consistent with the structural data that α-KG can bind tightly to the active site of NcT7H. In the α-KG-bound NcT7H structure, the C3 atom of α-KG is about 3.6 Å away from the hydroxyl group of Tyr194. When NOG is docked into the active site, the N3 atom of NOG could form a hydrogen bond with the side chain of Tyr194 to strength its binding, explaining the higher affinity for NOG.

**Table 2.** ITC measured thermodynamic parameters of NcT7H with different ligands

Ligand	*K*_d_ (μM)	ΔH (kcal/mol)	TΔS (kcal/mol)	n Value
α-KG	34.4 ± 6.63	−6.03 ± 0.56	0.06	0.80 ± 0.05
NOG	5.35 ± 1.26	−5.14 ± 0.20	2.05	1.04 ± 0.03
Succinate	ND	ND	ND	ND
U	ND	ND	ND	ND
T	32.0 ± 5.24	−8.45 ± 0.74	-2.32	0.52 ± 0.03
5hmU	30.5 ± 5.59	−10.8 ± 1.19	-4.59	0.45 ± 0.04
5fU	108 ± 13.0	−8.73 ± 0.60	-3.31	0.20 ± 0.00
5caU	ND	ND	ND	ND

The protein used for analysing the binding affinity of NcT7H for U, T, 5hmU, 5fU and 5caU was pre-incubated with the cosubstrate analogue NOG. ND, not detected.

In the absence of α-KG or NOG, NcT7H has no detectable binding with all the substrates (data not shown). In the presence of NOG (substituting α-KG to avoid catalytic reaction), NcT7H has moderate binding affinities for T and 5hmU (*K*_d_ of 32.0 ± 5.24 μM and 30.5 ± 5.59 μM, respectively) and a 3-fold lower affinity for 5fU (*K*_d_ of 108 ± 13.0 μM), but has no detectable binding with 5caU and U (Figure 3 and Table 2). These results indicate that NcT7H binds the substrates only in the presence of α-KG, which is consistent with the previous biochemical and structural studies of other α-KG dependent dioxygenases (44). Moreover, these results suggest that the C5 modification groups of the substrates play some roles in determining the substrate specificity and in the substrate binding.

Furthermore, our ITC results show that NcT7H has no detectable binding with the α-KG product succinate (Figure 3 and Table 2) and in the presence of succinate, NcT7H also has no detectable binding with the substrates (data not shown), consistent with our crystallization experiment results that NcT7H yielded no crystals in the presence of succinate alone or succinate and 5hmU, 5fU or 5caU. These data suggest that after each oxidation reaction, the products (succinate and 5hmU, 5fU or 5caU) are released from the enzyme, and then new α-KG and substrate are reloaded for next oxidation reaction.

### Biochemical and mutagenesis analyses

**Figure 4.**
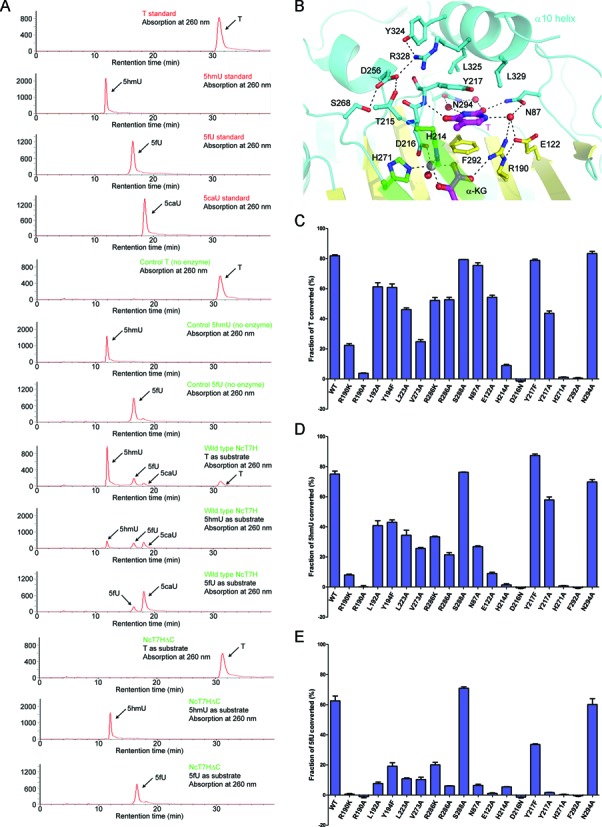
Apparent enzymatic activities of wild-type and mutant NcT7H. (**A**) Activities of the full-length of NcT7H and the C-terminal truncated NcT7HΔC for the oxidation of T, 5hmU and 5fU analysed with the HPLC method. The standard T, 5hmU, 5fU and 5caU were used as references. (**B**) Interactions of the α10 helix with the residues composing the active site in the structure of NcT7H-T complex. The key residues, the substrate and α-KG are shown with ball-and-stick models. The hydrogen bonds are indicated with black dotted lines. (**C–E**) The effects of mutations of the key residues at the active site on the apparent enzymatic activity for the oxidation of (C) T, (D) 5hmU and (E) 5fU, respectively.

We first characterized the apparent enzymatic activities of NcT7H towards different substrates using the HPLC method (26) (Figure 4A). At the standard condition, about 80% of T was oxidized to 5hmU, 5fU and 5caU with gradually decreasing amounts; and about 80% of 5hmU was converted to 5fU and 5caU with approximately equal amounts. As the catalytic reaction towards 5fU is faster than that towards T and 5hmU, the amount of enzyme used in the assay was 2.5 fold less. At that condition, about 60% of 5fU was converted to 5caU.

We also performed kinetic studies to measure the *K*_m_ and *k*_cat_ values of NcT7H towards different substrates using the SCS/PK/LDH-coupled system (36). NcT7H exhibits comparable *K*_m_ and *k*_cat_ values towards T and 5hmU but displays about 1.8-fold higher *K*_m_ value and 2-fold higher *k*_cat_ value towards 5fU, and the catalytic efficiency of NcT7H towards different substrates is in the order of 5fU ≥ T >5hmU (Table 3 and Supplementary Figure S6), which are consistent with our binding affinity and activity assay results (Figures 3 and 4, and Table 2). However, it should be noted that at the assay condition, the catalytic reaction for 5fU is faster than that for T and 5hmU and 5fU is also less stable than T and 5hmU, and thus the variation of the velocity for 5fU is much higher than that for T and 5hmU. This might explain that the kinetic parameters reported in this study and the previous studies of *Rhodotorula glutinis* T7H (27,28) are comparable for T and 5hmU but vary greatly for 5fU.

**Table 3.** Kinetic parameters of wild-type and mutant NcT7H towards different substrates

	Substrate	*K*_m_ (μM)	*k*_cat_ (min^−1^)	*k*_cat_/*K*_m_ (min^−1^·μM^−1^)
WT	T	141 ± 13.4	25.0 ± 0.74	0.177
	5hmU	178 ± 16.4	19.5 ± 0.58	0.110
	5fU	253 ± 32.2	45.9 ± 2.14	0.181
R190K	T	542 ± 64.7	3.30 ± 0.18	0.006
	5hmU	1016 ± 200	2.12 ± 0.23	0.002
	5fU	ND	ND	ND
R286A	T	649 ± 74.1	24.3 ± 1.29	0.037
	5hmU	950 ± 114	8.46 ± 0.55	0.009
	5fU	ND	ND	ND
N87A	T	638 ± 128	13.8 ± 1.32	0.022
	5hmU	1065 ± 156	5.77 ± 0.48	0.005
	5fU	ND	ND	ND
Y217A	T	1500 ± 332	15.4 ± 2.13	0.010
	5hmU	2062 ± 360	25.6 ± 3.02	0.012
	5fU	ND	ND	ND
Y217F	T	128 ± 18.5	32.5 ± 1.37	0.254
	5hmU	247 ± 35.2	33.2 ± 1.68	0.134
	5fU	718 ± 213	14.2 ± 2.11	0.020

ND, not detected.

In the full-length NcT7H structures, the C-terminal region forms a long helix (α10) that covers on top of the substrate-binding pocket (Figure 4B). Although helix α10 does not directly interact with α-KG and substrate, it contributes to the formation of the substrate-binding pocket. Specifically, several residues of helix α10 (particularly Arg328) stabilize the side chain of Tyr217 which makes hydrophobic interactions with the substrate. In addition, the strictly conserved residues Tyr324 and Arg328 of helix α10 are involved in a hydrogen-bonding network with three highly conserved residues (Thr215, Asp256 and Ser268) located adjacent to the HXD/E…H motif. These interactions would stabilize the conformations of the structure elements at the active site and in turn constrain the conformation of helix α10 to form a closed substrate-binding pocket (Figure 4B). Indeed, our crystallization experiments show that co-crystallization of NcT7HΔC with α-KG and the substrates yielded only the α-KG-bound form (data not shown). In addition, our biochemical data show that NcT7HΔC could not catalyse the oxidation reaction of T, 5hmU or 5fU (Figure 4A). The structural and biochemical data together indicate that the C-terminal region of NcT7H is essential for the substrate binding and catalysis.

To examine the functional roles of key residues at the active site in the catalytic reaction, we performed mutagenesis and biochemical studies (Figure 4C–E). Single mutation of any of the three residues involved in the metal ion binding (His214, Asp216 and His271) results in almost complete loss of the activity towards all substrates as these mutations would affect the Fe^2+^ binding which is essential for catalysis. Among the residues involved in the α-KG binding, Arg190 has hydrogen-bonding interactions with the C1-carboxylate of α-KG and the C5-hydroxymethyl group of 5hmU (or the C5-formyl group of 5fU), and thus its mutation to Lys or Ala leads to drastic loss of the activity towards all substrates (Figure 4C–E). Mutations of the other residues (Leu192, Tyr194, Leu223, Val273, Arg286 and Ser288) moderately decrease the activity towards T, 5hmU and 5fU with gradually increasing effects in the order of T< 5hmU< 5fU. These results indicate that the activity of NcT7H towards T, 5hmU and 5fU has an increasing dependency on the proper binding of α-KG.

Among the residues involved in the substrate binding, the side chain of Phe292 has π–π interaction with the substrates and mutation F292A abolishes the activity towards all substrates. The side chain of Tyr217 makes hydrophobic interactions with the substrates; mutation Y217F has insignificant effects on the activity towards T and 5hmU but a moderate effect on the activity towards 5fU, and mutation Y217A has moderate effects on the activity towards T and 5hmU but abolishes the activity towards 5fU. Sequence alignment shows that Phe292 is strictly conserved, and Tyr217 is highly conserved but can be substituted by Phe or Trp in some species (Supplementary Figure S4). As residues Asn87, Glu122 and Asn294 are mainly involved in water molecule mediated hydrogen-bonding interactions with the substrate, mutations N87A and E122A do not severely impair the activity towards T but can dramatically reduce the activity towards 5hmU and 5fU, and mutation N294A does not affect the activity towards all substrates. These results indicate that mutations of the residues participating in the substrate binding affect the activity to varied extents with gradually increasing effects in the order of T< 5hmU< 5fU as well.

Furthermore, we carried out mutagenesis and kinetic studies to verify the functional roles of several key residues at the active site (Table 3 and Supplementary Figure S6). Mutations N87A, R190K and R286A have moderate effects on the *K*_m_ value (increased by 4–6 folds) and varied effects on the *k*_cat_ value (decreased by 1–9 folds) towards T and 5hmU, and the catalytic efficiencies of these mutants are substantially decreased by 5–55 folds. Mutation Y217A substantially increases the *K*_m_ value (11–12 folds) towards T and 5hmU but has minor effect on the *k*_cat_ value, and thus the catalytic efficiency of this mutant is decreased by 18 folds and 9 folds, respectively. Additionally, these mutations abolish the activity for 5fU, leading to undetectable *K*_m_ and *k*_cat_ values. Interestingly, mutation Y217F has minor effects on the *K*_m_ value but causes a slightly increased *k*_cat_ value for T and 5hmU, leading to a slightly increased catalytic efficiency. However, this mutation has moderate effects on both the *K*_m_ (increased by 3 folds) and *k*_cat_ values (decreased by 3 folds) for 5fU, and thus the Y217F mutant has a substantially decreased catalytic efficiency towards 5fU. These results suggest that the hydroxyl group of Tyr217 has differed effects on the catalytic reaction for different substrates. Overall, the kinetic results are in good agreement with the activity assay results (Figure 4C–E).

Taken together, our structural and biochemical data show that His214, Asp216 and His271 play essential roles in the metal ion binding; Arg286 plays an important role in the binding of α-KG; Arg190 plays a vital role in the binding of both α-KG and the substrate, and Phe292 and Tyr217 play critical roles in the substrate binding. The other residues at the active site have varied effects on the binding of α-KG and the substrate. The proper binding of α-KG and the substrate has gradually increasing effects on the activity towards the substrates in the order of T< 5hmU< 5fU.

### Substrate specificity and catalytic mechanism of NcT7H

Our structural and biochemical data demonstrate that NcT7H can bind T, 5hmU and 5fU with slightly differed binding affinity only in the presence of α-KG but can not bind U and 5caU (Figure 3 and Table 2). In the substrate-bound NcT7H structures, the substrates maintain almost identical interactions with the enzyme, including the hydrophobic interactions with the side chains of Phe292 and Tyr217 and the hydrogen-bonding interactions with several conserved residues (Figure 2C–E). These interactions play important roles in the substrate binding as mutations of these residues have severe to moderate effects on the substrate binding and the activity (Figure 4C–E, Table 3 and Supplementary Figure S6). As the C5 modification groups of T, 5hmU and 5fU make slightly differed interactions with α-KG and the enzyme, they might play important roles in the substrate binding and differentiation (Figure 3 and Table 2). Presumably, the differed C5 modification groups would have varied effects on the chemical property of the uracil moiety and thus could affect its interactions with the enzyme. Furthermore, the strictly conserved Arg190 makes interactions with both α-KG and the substrate and thus also plays a critical role in the substrate recognition and the catalytic reaction (Figure 2D and E, Figure 4C–E, Table 3 and Supplementary Figure S6). These factors together allow NcT7H to distinguish the substrates T, 5hmU and 5fU from U and 5caU, and to determine the slightly varied binding affinity and/or enzymatic activity towards different substrates.

Previous structural and biochemical studies of other α-KG dependent dioxygenases have shown that α-KG binds to the enzyme with either ‘in-line’ or ‘off-line’ mode and consequently there are two slightly differed catalytic mechanisms (37,43,44). In the substrate-bound NcT7H structures, the C1-carboxylate and C2-oxo groups of α-KG are located in opposite to His271 and Asp216 of the HXD/E…H motif, respectively, and Wat1 (which occupies presumably the oxygen in catalysis) is located below the plane formed by the C1-carboxylate of α-KG, Asp216 and His271 and in opposite to His214 (Figure 2B–E), indicating that the α-KG binding assumes the ‘off-line’ mode in NcT7H. As our structural and biochemical data have identified the key residues involved in the binding of the metal ion, α-KG and the substrate and their functional roles in the catalysis, we can propose a detailed catalytic mechanism for NcT7H, which is very similar to that for other α-KG dependent dioxygenases with the ‘off-line’ α-KG binding mode (44) (Supplementary Figure S7).

## DISCUSSION

The TET proteins can catalyse the consecutive oxidations of 5mC to 5hmC, 5fC and 5caC in active DNA demethylation in mammals, and play critical roles in epigenetic regulation (11–14). Although the crystal structures of the HsTET2-DNA and NgTET1-DNA complexes have been reported (16,17), the molecular basis for how the TET proteins recognize different C5 modification groups on cytosine and catalyse the consecutive oxidations are still elusive. As the conversion of T to 5hmU, 5fU and 5caU catalysed by T7H in fungi is chemically similar to that of 5mC to 5hmC, 5fC and 5caC catalysed by the TET proteins in mammals, we carried out the structural and biochemical studies of NcT7H to investigate the molecular basis for the substrate specificity and catalytic mechanism of T7H, hoping that this knowledge could advance our understanding of the structure and function of the TET proteins. Indeed, structural comparison of NcT7H with other α-KG dependent dioxygenases and in particular the TET proteins provides new insights into the molecular mechanism of the substrate recognition and catalytic reaction of the TET proteins.

As expected, structural similarity search using the Dali server (45) shows that the structure of NcT7H is similar to several α-KG dependent dioxygenases, including HsTET2, NgTET1, *Aspergillus nidulans* isopenicillin N synthase (AnIPNS), *H. sapiens* AlkB homology 5 (HsAlkBH5), *E. coli* AlkB (EcAlkB) and *H. sapiens* FTO (HsFTO). The DSBH core of these enzymes can be superimposed well albeit the flanking structure elements are much different (Supplementary Table S2 and Figure S8). Particularly, despite of the low sequence identity, the HXD/E…H motif which is involved in the metal ion binding and the Arg residue which is involved in stabilization of the C5-carboxylate of α-KG (corresponding to Arg286 in NcT7H) in these enzymes, the common features of the α-KG dependent dioxygenases (37,40), could be structurally aligned very well (Supplementary Figures S9 and S10).

**Figure 5.**
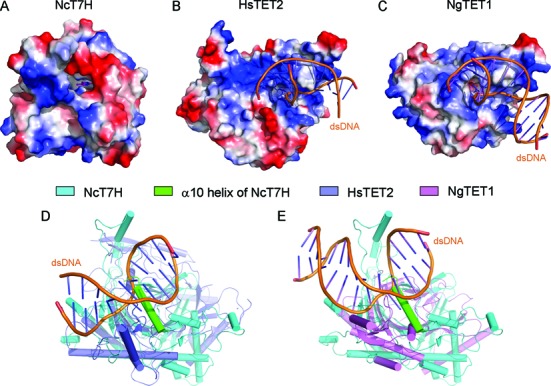
Structural comparison of NcT7H with the TET proteins. (**A–C**) Electrostatic potential surfaces of (A) NcT7H, (B) HsTET2 and (C) NgTET1. The surface charge distribution is displayed as blue for positive, red for negative and white for neutral. (**D**) Structural comparison of NcT7H with HsTET2-DNA. (**E**) Structural comparison of NcT7H with NgTET1-DNA. The bound dsDNA are shown with yellow ribbons.

Nevertheless, a detailed structural comparison of NcT7H, HsTET2 and NgTET1 shows that there are substantial structural differences at the active site. The active sites of HsTET2 and NgTET1 are relatively open, whereas the active site of NcT7H is relatively closed because the C-terminal α10 helix covers on top of the active site and it would cause steric conflict with the bound DNA in the HsTET2-DNA and NgTET1-DNA complexes (Figure 5). Besides, the surface surrounding the active site in HsTET2 and NgTET1 is largely positively charged (Figure 5B and C), while that in NcT7H is largely negatively charged which is unfavourable for binding DNA (Figure 5A). These differences may determine that the active site of NcT7H binds only a free base but not a modified nucleotide in DNA.

Furthermore, the structural comparison also reveals that the recognition and binding manners of the bases have some commonalities and differences between NcT7H and the TET proteins. As the substrate of NcT7H is a free base, the substrate is sandwiched by two aromatic residues (Phe292 and Tyr217) to stabilize its binding (Figure 2C–E). However, as the substrate of the TET proteins is a modified nucleotide in DNA and its conformation is constrained by the DNA, only one side of 5mC is involved in hydrophobic interactions with an aromatic residue (Tyr1902 of HsTET2 and Phe295 of NgTET1) (Supplementary Figure S9A and C). In addition, the substrates of both NcT7H and the TET proteins make hydrogen-bonding interactions with several residues either directly or indirectly via water molecules; however, these involved residues are not very conserved (Supplementary Figures S9B and D and S10). On the other hand, the interactions of α-KG with the proteins are conserved in all these structures (Figure 2B and Supplementary Figure S9A–D). Specifically, the C1-carboxylate and C5-carboxylate of α-KG are each stabilized by an Arg residue (Arg190 and Arg286 of NcT7H, Arg1261 and Arg1896 of HsTET2, and Arg224 and Arg289 of NgTET1, respectively) via hydrogen-bonding interactions albeit the Arg residue interacting with the C1-carboxylate is provided by different structure elements (Supplementary Figure S10).

As Phe292 and Tyr217 of NcT7H play important roles in the substrate binding, we also compared the active site of NcT7H with that of EcAlkB and HsFTO, two other dioxygenases with nucleic acid substrates. Similar to that in NcT7H, the substrate base in EcAlkB and HsFTO is also sandwiched by two large side-chain residues (Trp69 and His131 in EcAlkB and Tyr108 and His231 in HsFTO) (Supplementary Figure S9E–H). However, these residues are contributed by different structural elements and thus the binding orientation of the substrate base in EcAlkB and HsFTO is different from that in NcT7H and the TET proteins. These results indicate that stabilization of the substrate base by large side-chain residue(s) is a common feature of these dioxygenases.

Our structural and biochemical data have demonstrated that the conserved Arg190 of NcT7H plays a critical role in the binding of α-KG and the substrate and the catalytic reaction. In the substrate-bound NcT7H structures, the side chain of Arg190 makes hydrogen-bonding interaction and/or van der Waals contacts with both the C1-carboxylate of α-KG and the C5 modification group of the substrate (Figure 2C–E). In the HsTET2-DNA and NgTET1-DNA structures, there is a conserved Arg residue (Arg1261 of HsTET2 and Arg224 of NgTET1) from a different structure element, which occupies a similar spatial position as Arg190 of NcT7H and has hydrogen-bonding interaction with the C1-carboxylate of α-KG. However, secondary structural alignment shows that Arg190 of NcT7H is equivalent to Thr1372 of HsTET2 or Ala212 of NgTET1 (Supplementary Figure S10). These residues are located on a β-strand (corresponding to β7 of NcT7H) which is proposed to be involved in substrate binding (37). In the HsTET2-DNA and NgTET1-DNA structures, Thr1372 of HsTET2 and Ala212 of NgTET1 make van der Waals contacts with the 5-methyl group of 5mC and thus might play some roles in the recognition of the 5-methyl group of 5mC (16,17). These results suggest that HsTET2 and NgTET1 might use two residues to recognize and bind the C1-carboxylate of α-KG and the C5 modification group of the substrate, respectively, whereas NcT7H uses a single Arg190 to exert the dual functions.

Our structural and biochemical data of NcT7H show that the products are released after each oxidation reaction and new cosubstrate and substrate are reloaded to conduct the next oxidation reaction. In the HsTET2 and NgTET1 structures, the 5mC is flipped out of the DNA and inserted into the active site, and α-KG is deeply buried at the active site (16,17). Although the DNA blocks the entrance to the active site, it has extensive interactions with the protein and thus its dissociation from the protein might be difficult. Hence, there might be two possibilities for the TET proteins to reload a new α-KG and carry out the next oxidation reaction. One possibility is that after each oxidation reaction, the DNA is released from the protein to unblock the entrance to the active site, allowing release of succinate and reloading of a new α-KG. In this case, the consecutive oxidations of 5mC by the TET proteins is also discontinuous, and thus the oxidized intermediates of 5mC might have specific functions and should be regulated precisely, which is in agreement with the previous studies showing that the oxidation products of 5mC could be positioned at different regulatory regions and contribute to different transcriptional states (46,47). It is also possible that after each oxidation reaction, the DNA is not dissociated from the protein; instead, the active site undergoes conformational changes to release succinate and then reload a new α-KG. In this case, the TET proteins could catalyse the consecutive oxidations of 5mC to 5caC continuously and thus the oxidized intermediates of 5mC might have less functional roles. Further structural and functional studies of the TET proteins are needed to resolve this issue.

## ACCESSION NUMBERS

The crystal structures of the apo NcT7HΔC and the full-length NcT7H in complexes with α-KG, with α-KG and T, with α-KG and 5hmU, and with α-KG and 5fU have been deposited in the Protein Data Bank under accession codes 5C3O, 5C3P, 5C3Q, 5C3R, and 5C3S, respectively.

## SUPPLEMENTARY DATA

Supplementary Data are available at NAR Online.

SUPPLEMENTARY DATA
