# Supplementary material for: Molecular basis for the substrate specificity and catalytic mechanism of thymine-7-hydroxylase in fungi
Source: Nucleic Acids Res. 2015 Oct 1;43(20):10026–38. doi: 10.1093/nar/gkv979 (PMC4787775; doi:10.1093/nar/gkv979)
Supplement: SUPPLEMENTARY DATA [file supp_43_20_10026__index.html]

Molecular basis for the substrate specificity and catalytic mechanism of thymine-7-hydroxylase in fungi — Molecular basis for the substrate specificity and catalytic mechanism of thymine-7-hydroxylase in fungi — SUPPLEMENTARY DATA 

# Molecular basis for the substrate specificity and catalytic mechanism of thymine-7-hydroxylase in fungi

## SUPPLEMENTARY DATA

- SUPPLEMENTARY DATA
