# Supplementary material for: Molecular basis for the substrate specificity and catalytic mechanism of thymine-7-hydroxylase in fungi
Source: Nucleic Acids Res. 2015 Oct 1;43(20):10026–38. doi: 10.1093/nar/gkv979 (PMC4787775; doi:10.1093/nar/gkv979)
Supplement: SUPPLEMENTARY DATA [file supp_gkv979_nar-01785-f-2015-File007.docx]

**Supplementary Information**

**Supplementary Table S1. Inductively coupled plasma atomic emission spectroscopy results.**

| **Metal** | **NcT7HΔC solution (%)** | **Buffer 1 (%)** |
| --- | --- | --- |
| Ca | 0.0044 | 0.0018 |
| Mg | 0.0002 | 0.0002 |
| Ni | <0.0001 | <0.0001 |
| **Metal** | **NcT7H solution (%)** | **Buffer 2 (%)** |
| Ni | 0.0078 | 0.0042 |
| Ca | 0.0018 | 0.0012 |
| Mg | <0.0001 | <0.0001 |

Buffer 1: 20 mM Tris-HCl (pH 8.0) and 100 mM NaCl. Buffer 2: 20 mM Tris-HCl (pH 8.0), 100 mM NaCl, and 2 mM NiCl_2_.

**Supplementary Table S2. RMSD values between NcT7H and other α-KG dependent dioxygenases.**

| **Dioxygenase** | **RMSD (Å)** | **Numbers of aligned Cα** |
| --- | --- | --- |
| HsTET2 | 3.2 | 167 |
| NgTET1 | 3.3 | 164 |
| AnIPNS | 1.8 | 256 |
| HsALKBH5 | 3.4 | 149 |
| EcAlkB | 3.2 | 135 |
| HsFTO | 3.2 | 173 |

Structural comparisons were performed based on the DSBH fold.

**Supplementary Figure Legends**

**Supplementary Figure S1.** Representative simulated annealing composite omit 2*Fo-Fc* maps (contoured at 1.0 σ level) for the metal ion, α-KG and the substrate at the active sites in the structures of (**A**) the NcT7H-AKG, (**B**) the NcT7H-T, (**C**) the NcT7H-5hmU, and (**D**) the NcT7H-5fU complexes.

**Supplementary Figure S2.** Topology of the full-length NcT7H. The minor and major β-sheets of the DSBH fold are colored in green and yellow, respectively. Other structure elements flanking the DSBH core are shown in cyan. The secondary structure elements are labeled.

**Supplementary Figure S3.** Comparison of the active site of different NcT7H structures. Comparison of the active site (**A**) in the NcT7HΔC (salmon) and NcT7H-AKG (green) structures, (**B**) in the NcT7H-AKG and NcT7H-T (cyan) structures, (**C**) in the NcT7H-T and NcT7H-5hmU (yellow) structures, (**D**) in the NcT7H-5hmU and NcT7H-5fU (magenta) structures, (**E**) in the NcT7H-T and NcT7H-5fU structures, and (**F**) in the NcT7H-T, NcT7H-5hmU and NcT7H-5fU structures. The residues, α-KG and the substrates are shown with ball-and-stick models. The divalent metal ion and water molecules are shown as spheres.

**Supplementary Figure S4.** Sequence alignment of T7Hs from different fungi species. The sequence number and secondary structures of NcT7H are placed on the top of the alignment. T7Hs from *Neurospora crassa*, *Rhodotorula glutinis*, *Aspergillus nidulans*, *Metarhizium robertsii*, *Metarhizium anisopliae*, *Neurospora tetrasperma*, *Colletotrichum orbiculare*, *Beauveria bassiana*, *Dichomitus squalens*, *Trametes versicolor*, *Aspergillus kawachii* are included in the alignment. Strictly conserved residues are highlighted in shaded red boxes and conserved residues in open red boxes. The key residues of NcT7H involved in the metal ion binding, the α-KG binding and the substrate binding are marked with blue stars, orange stars and green stars, respectively.

**Supplementary Figure S5.** Electrostatic potential surface of the active site in different NcT7H structures showing the α-KG and the substrate binding pockets. (**A**) NcT7H-AKG. (**B**) NcT7H-T. (**C**) NcT7H-5hmU. (**D**) NcT7H-5fU. The surface charge distribution is displayed as blue for positive, red for negative and white for neutral. α-KG, the substrates and the side chain of Arg190 are shown with ball-and-stick models. The metal ion is shown with a sphere.

**Supplementary Figure S6**. Kinetic analyses of wild-type and mutant NcT7H towards different substrates. All kinetic data were fitted to the Michaelis-Menten equation and all the experiments were performed in triplicates.

**Supplementary Figure S7**. Catalytic mechanism of NcT7H. 5hmU was used as the representative substrate. α-KG binds to NcT7H with the “off line” mode. Arg190 plays a key role in the binding of α-KG and the 5-hydroxymethyl group of 5hmU in the catalytic reaction. The bound O_2_ is first activated by Fe^II^ and then attacks the C2-oxo group of α-KG, leading to cleavage of the C1-C2 bond of α-KG to produce succinate and CO_2_ and formation of a ferryl-oxo (Fe^IV^=O or Fe^III^-O^-^) species (steps 4 and 5) ([1](#_ENREF_1),[2](#_ENREF_2)). After the release of CO_2_, the coordination of iron changes from the octahedral geometry into a distorted trigonal bipyramidal geometry, and the ferryl-oxo species moves closer to the C51 atom of the substrate and can abstract a hydrogen atom from the C51 atom of the substrate, leading to formation of the substrate radical and a Fe(III)-hydroxide species (steps 6 and 7). Finally, the substrate radical and the hydroxide join together to form the hydroxylated product (step 8). In the case of 5hmU as the substrate, dehydration of the hydroxylated product comes into 5fU (step 9). As the residues involved in the substrate recognition and binding and the catalytic reaction are strictly conserved, this catalytic mechanism should apply to T7Hs in other species.

**Supplementary Figure S8.** Structural comparisons of NcT7H with other α-KG dependent dioxygenases. (**A**) Structural comparison of NcT7H with the catalytic domain of HsTET2 (PDB code 4NM6). (**B**) Structural comparison of NcT7H with NgTET1 (PDB code 4LT5). (**C**) Structural comparison of NcT7H with *Aspergillus nidulans* IPNS (AnIPNS) (PDB code 1BLZ). (**D**) Structural comparison of NcT7H with *Homo sapiens* AlkB homology 5 (HsABH5) (PDB code 4NJ4). (**E**) Structural comparison of NcT7H with [*Escherichia coli*](javascript:void(0);) AlkB (EcAlkB) (PDB code 3BIE). (**F**) Structural comparison of NcT7H with the DSBH fold of *Homo sapiens* FTO (HsFTO) (PDB code 3LFM). The extra C-terminal structure elements of HsFTO are not included in the comparison. Superposition was performed based on the DSBH fold.

**Supplementary Figure S9.** Comparison of the active sites of NcT7H with other α-KG dependent dioxygenases. (**A**) Structure of the active site of HsTET2. (**B**) Comparison of the active site between NcT7H and HsTET2 (PDB code 4NM6). (**C**) Structure of the active site of NgTET1. (**D**) Comparison of the active site between NcT7H and NgTET1 (PDB code 4LT5). (**E**) Structure of the active site of EcAlkB. (**F**) Comparison of the active site between NcT7H and EcAlkB (PDB code 3BIE). (**G**) Structure of the active site of HsFTO. (**H**) Comparison of the active site between NcT7H and HsFTO (PDB code 3LFM).

**Supplementary Figure S10.** Structure-based sequence alignment of NcT7H, HsTET2, NgTET1 and several other α-KG dependent dioxygenases. The alignment was performed with the PROMALS3D server ([3](#_ENREF_3)). Secondary structure elements of NcT7H, HsTET2 and NgTET1 are placed on the top of the alignment. Strictly conserved residues are highlighted in shaded red boxes and conserved residues in open red boxes. The DSBH core of each protein is highlighted in yellow. Arg190 of NcT7H, Arg1261 of HsTET2 and Arg224 of NgTET1 are highlighted in green. The PDB codes of the structures used in the alignment are as follows: HsTET2 (4NM6), NgTET1 (4LT5), HsALKBH5 (4NJ4), EcAlkB (3BIE) and AnIPNS (1BLZ). The abbreviations for the species are as follows: Nc, *Neurospora crassa*; Hs, *Homo sapiens*; Ng, *Naegleria*; Ec, *Escherichia coli*; Tb, *Trypanosoma brucei brucei*; An, *Aspergillus nidulans*.

**References**

1. Hoffart, L.M., Barr, E.W., Guyer, R.B., Bollinger, J.M., Jr. and Krebs, C. (2006) Direct spectroscopic detection of a C-H-cleaving high-spin Fe(IV) complex in a prolyl-4-hydroxylase. *Proc. Natl. Acad. Sci. U. S. A.*, **103**, 14738-14743.

2. Bollinger, J.M., Price, J.C., Hoffart, L.M., Barr, E.W. and Krebs, C. (2005) Mechanism of taurine: alpha-ketoglutarate dioxygenase (TauD) from Escherichia coli. *Eur. J. Inorg. Chem.*, 4245-4254.

3. Pei, J., Kim, B.H. and Grishin, N.V. (2008) PROMALS3D: a tool for multiple protein sequence and structure alignments. *Nucleic Acids Res.*, **36**, 2295-2300.

**Supplementary Figure S1**

**
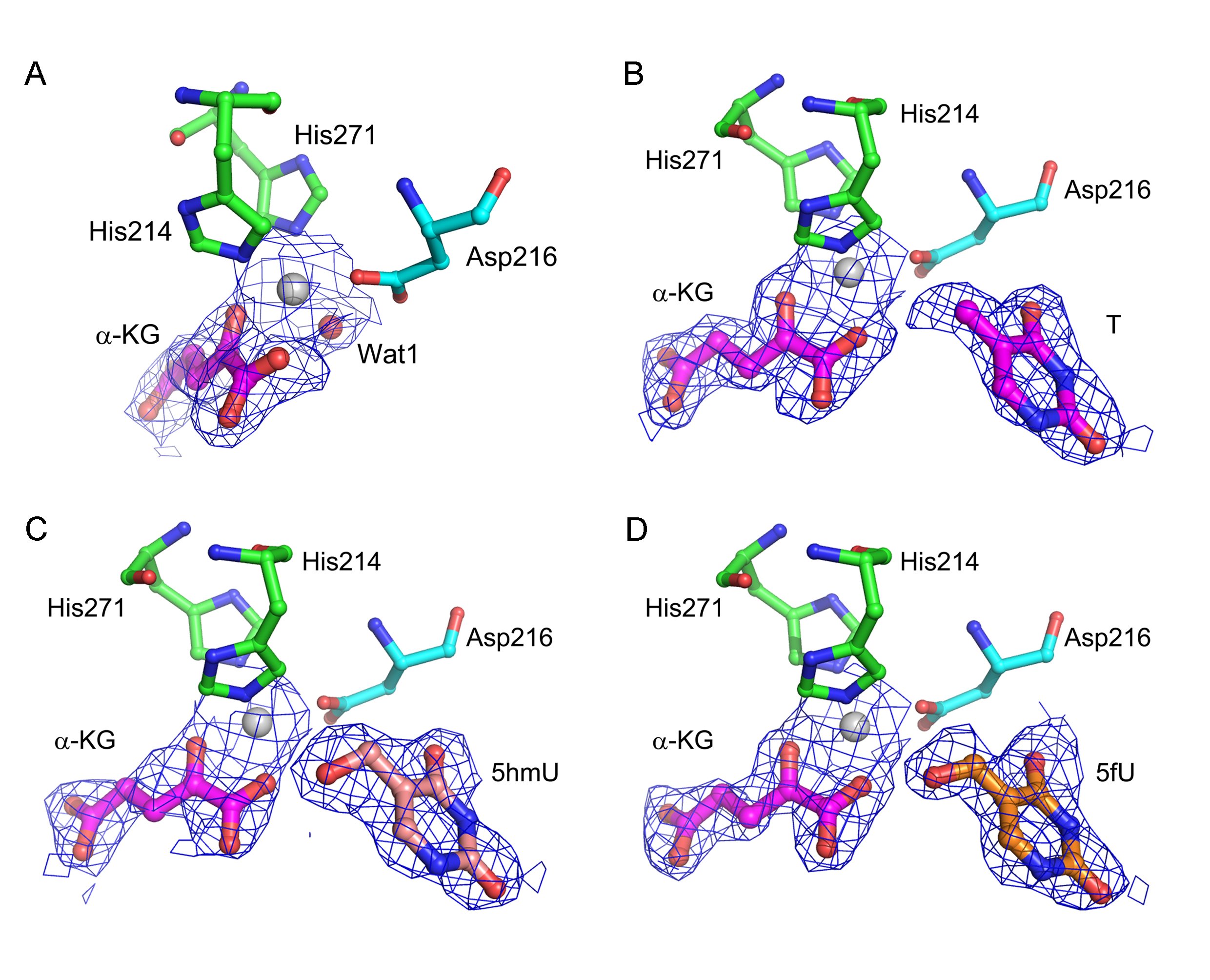
**

**Supplementary Figure S2**

**
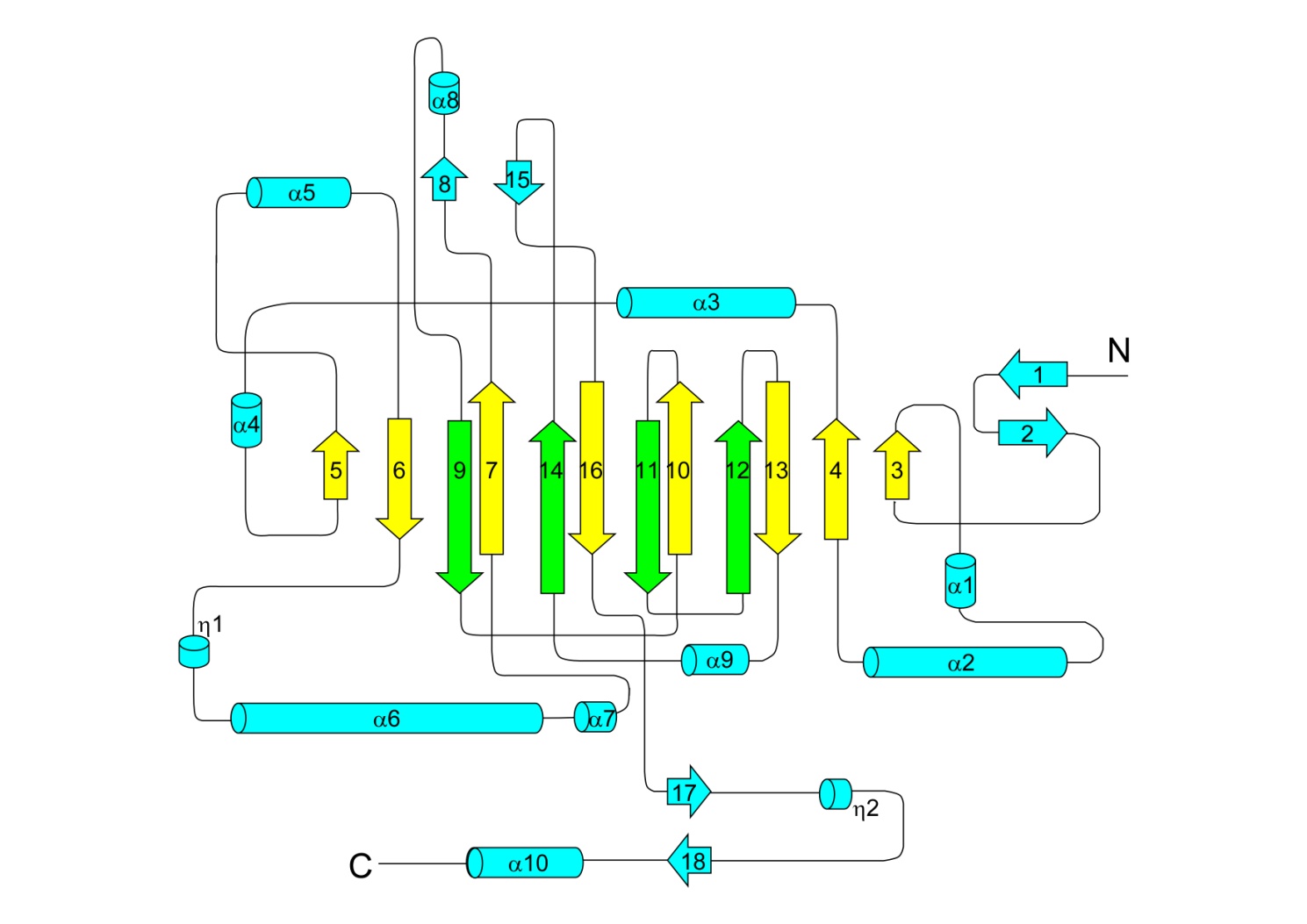
**

**Supplementary Figure S3**

**
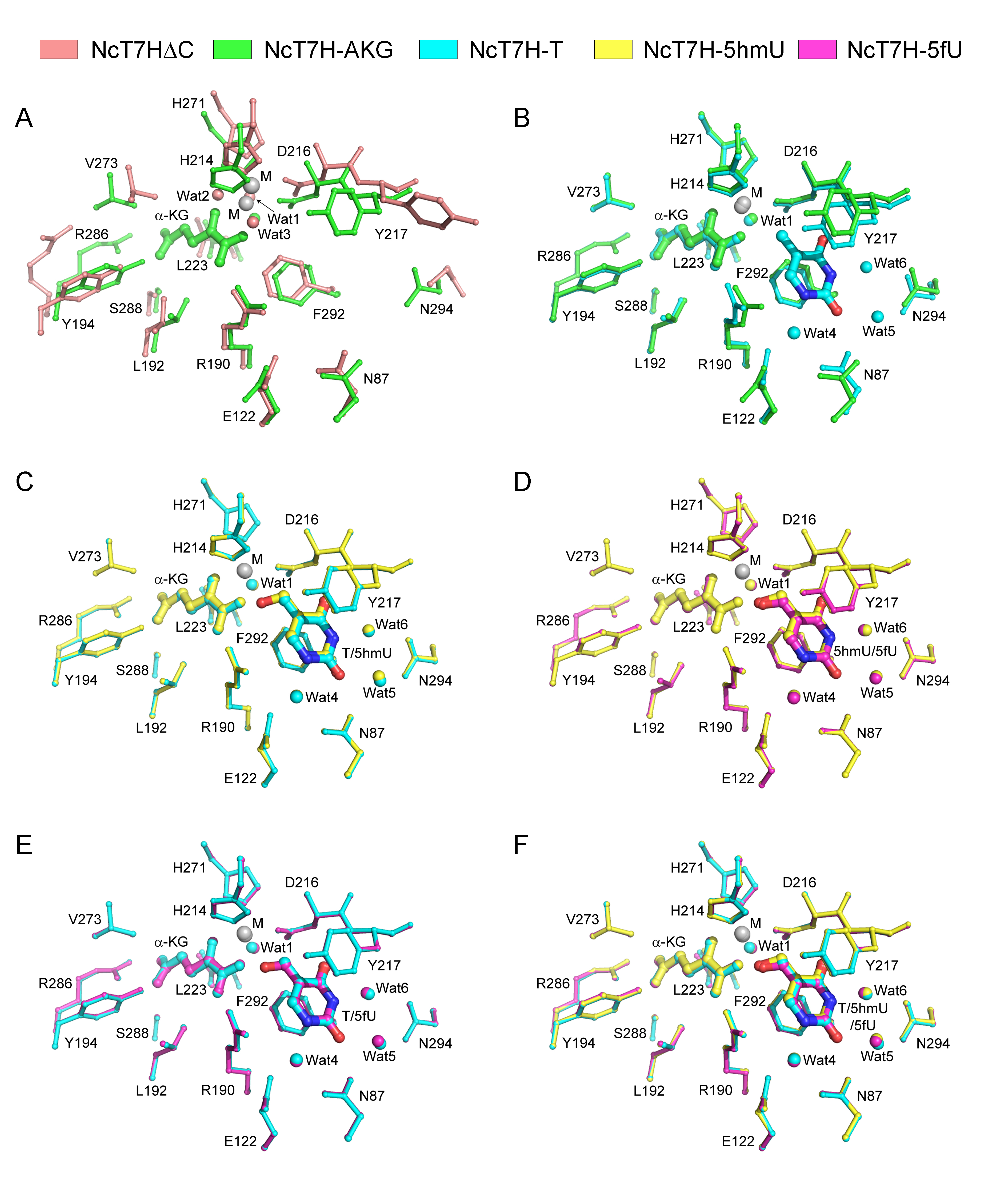
**

**Supplementary Figure S4**

**
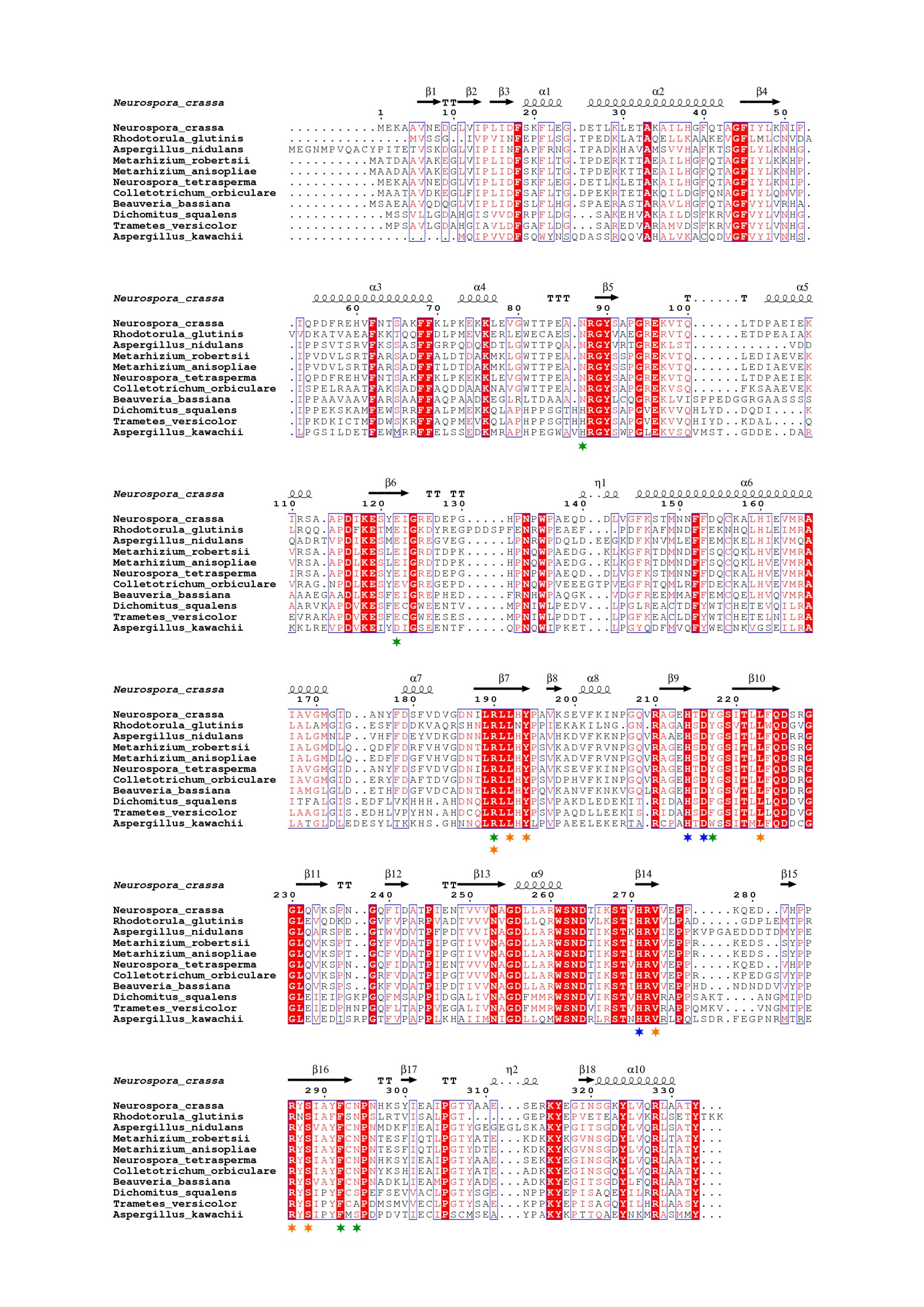
**

**Supplementary Figure S5**

**
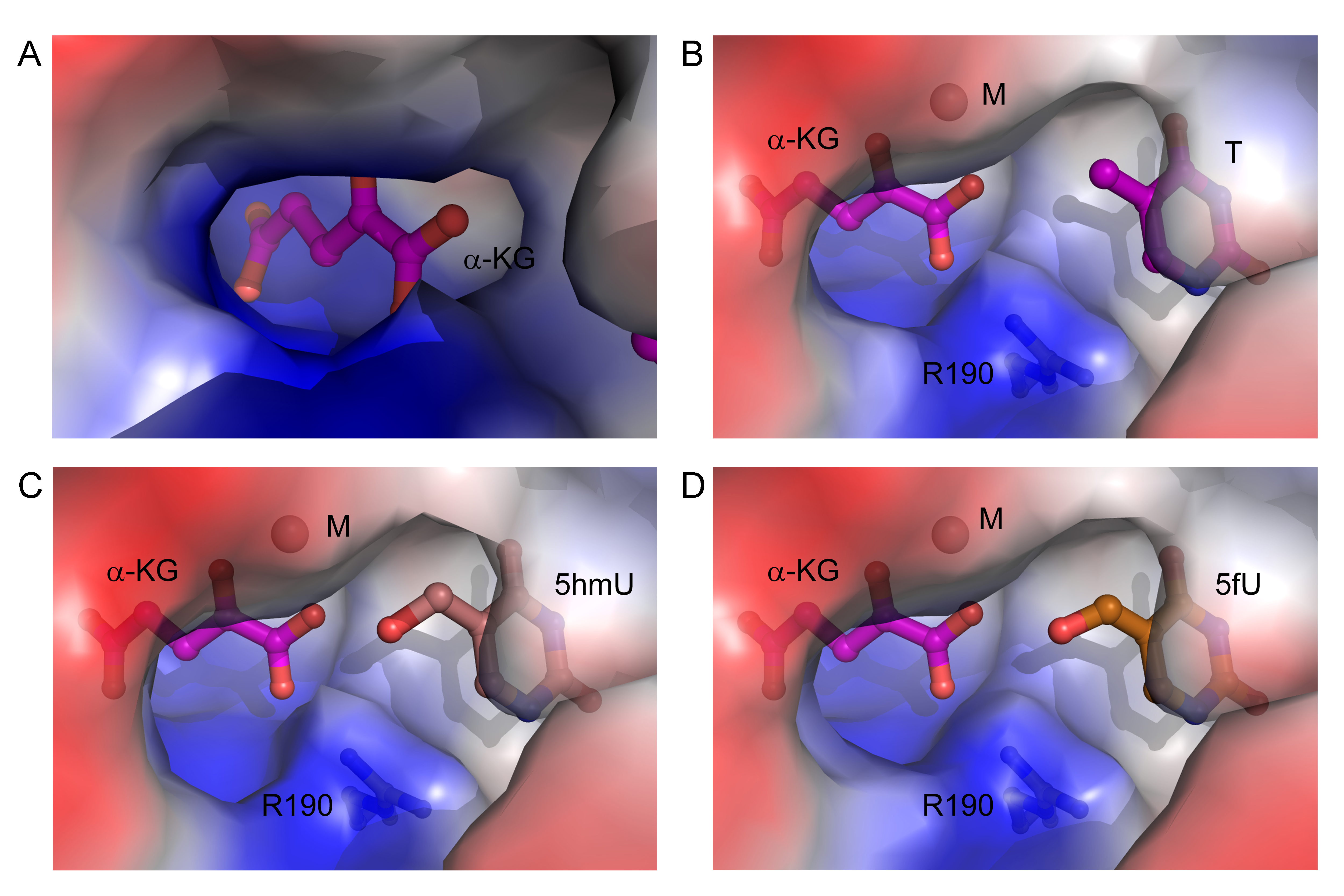
**

**Supplementary Figure S6**

**
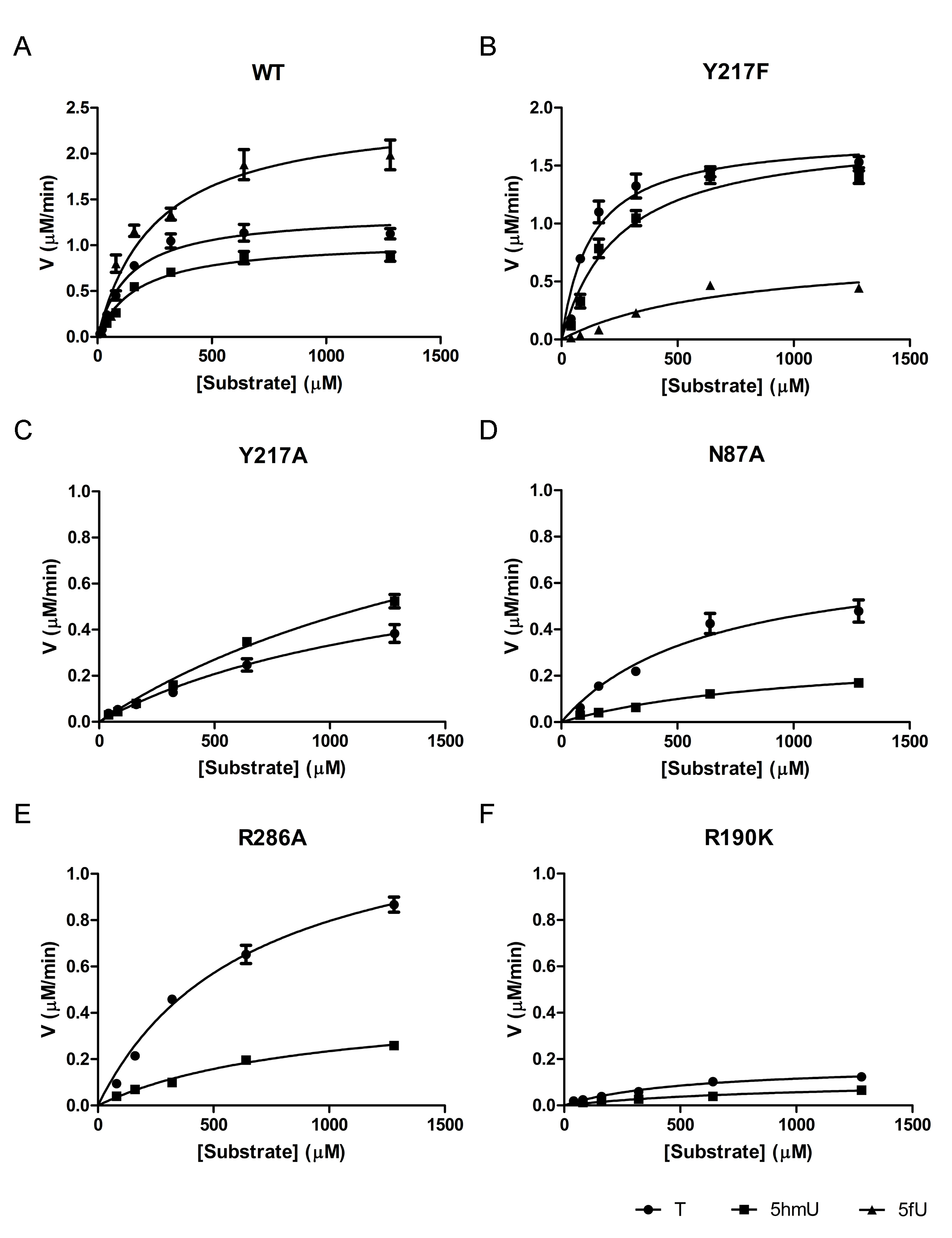
**

**Supplementary Figure S7**

**
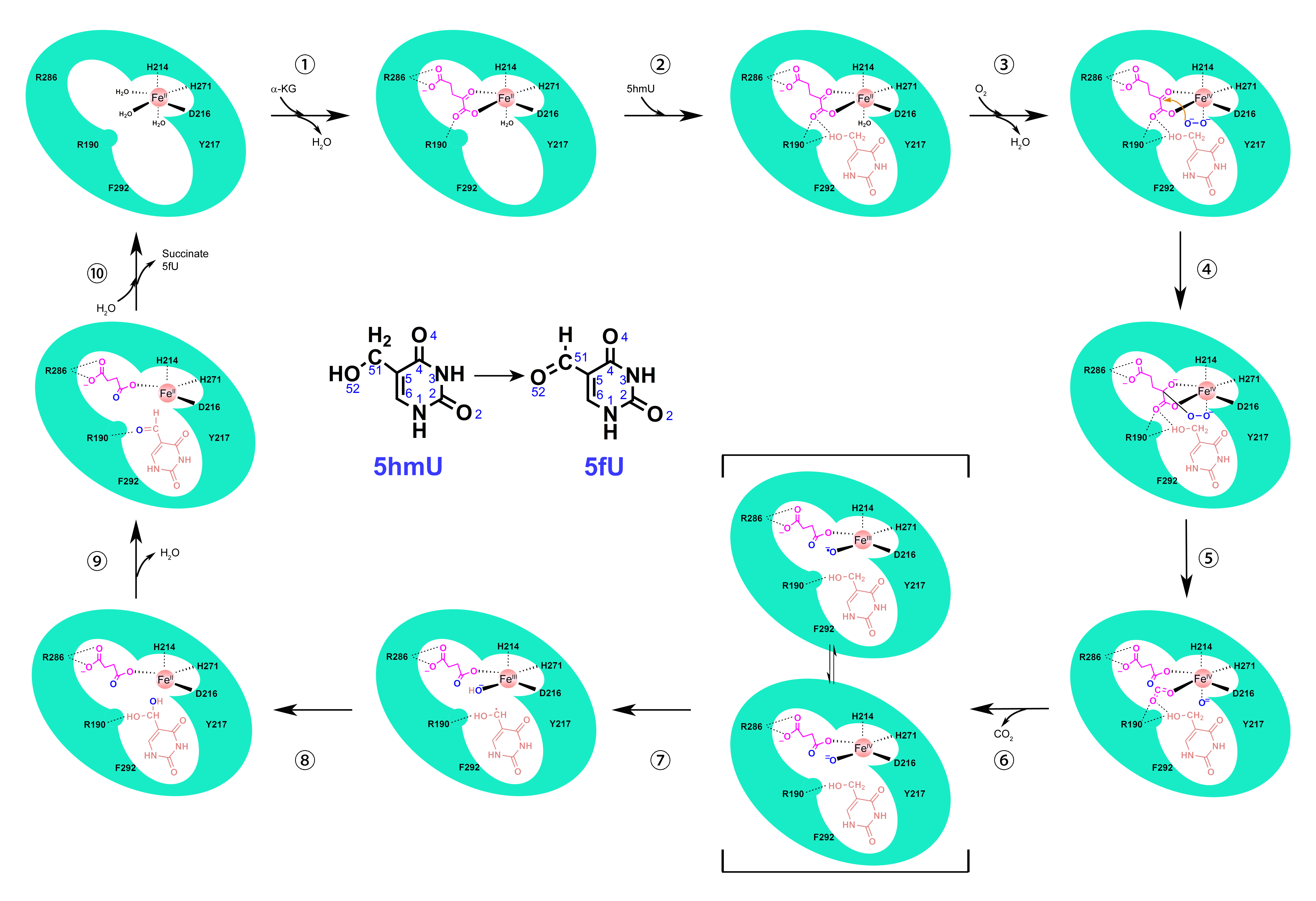
**

**Supplementary Figure S8**

**
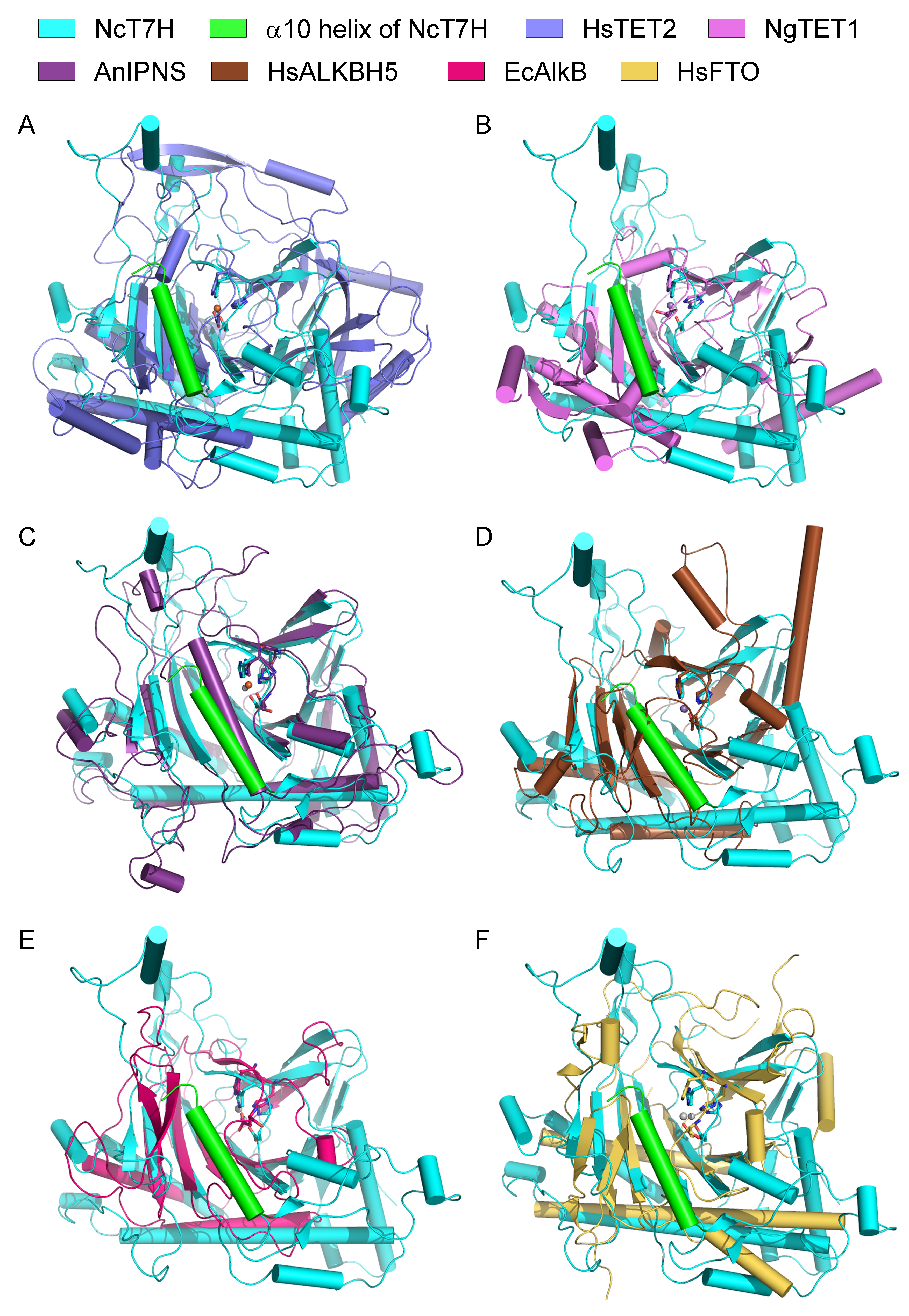
**

**Supplementary Figure S9**

**
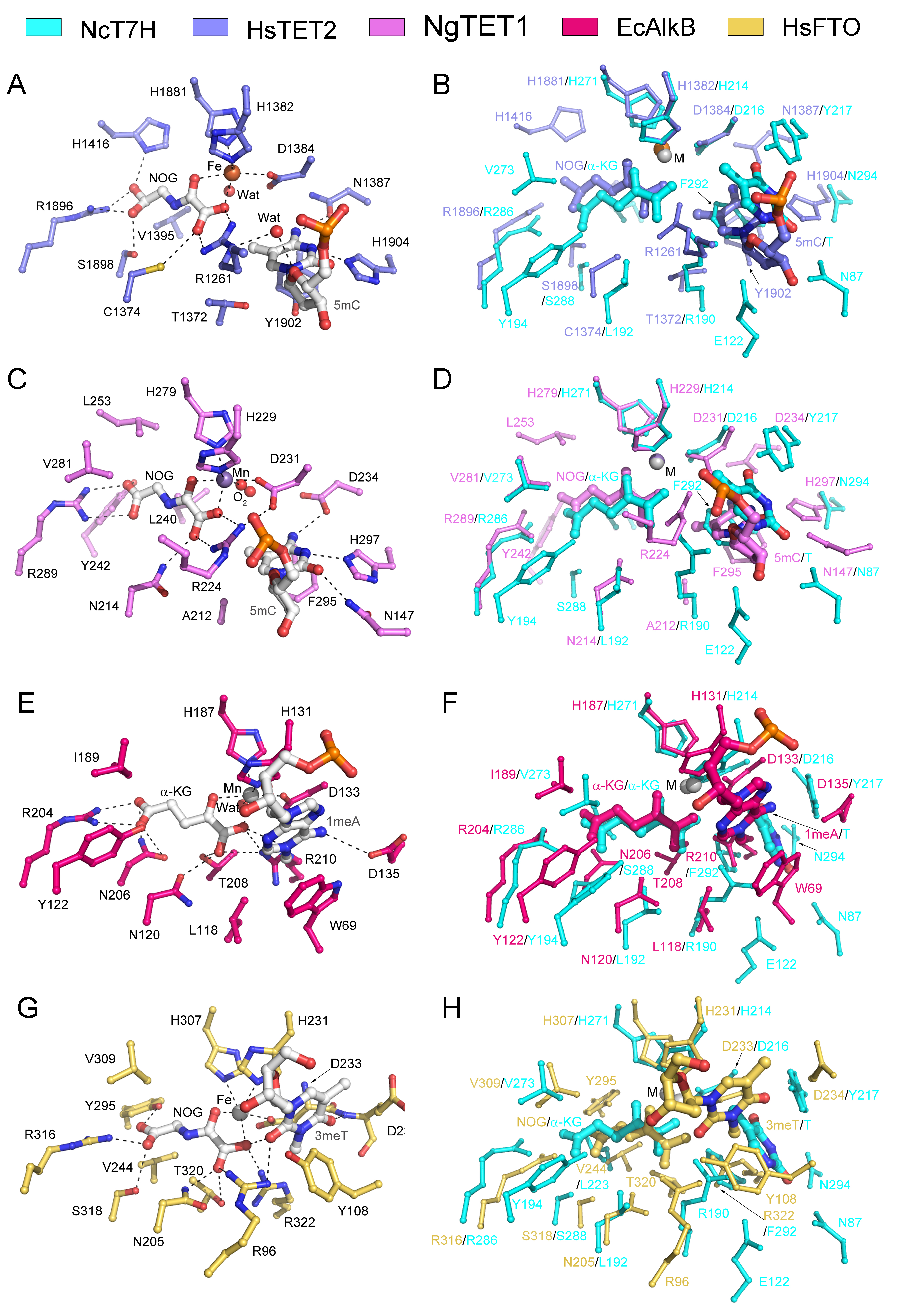
**

**Supplementary Figure S10**

**
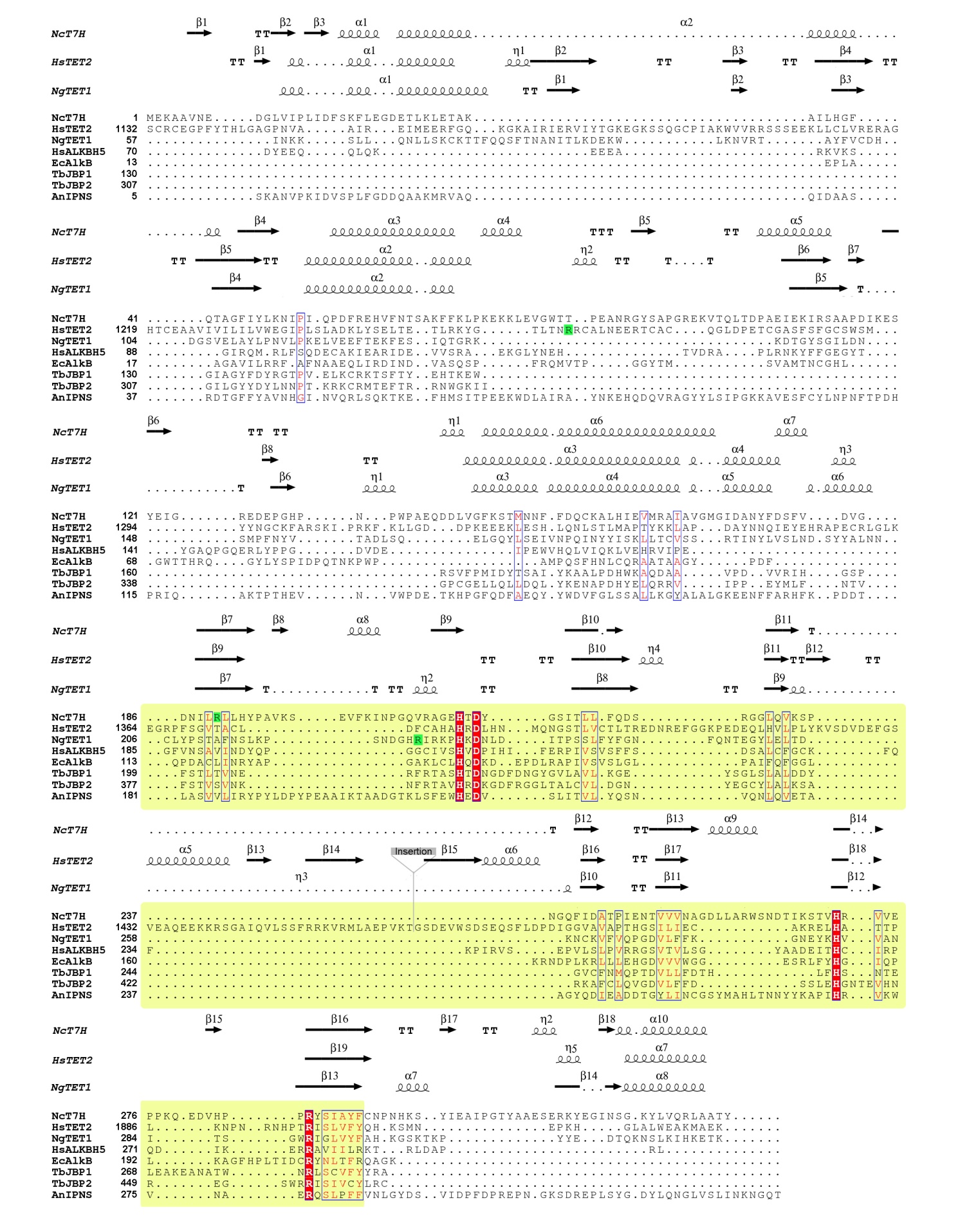
**
